# A Mild Method for Preparation of Highly Selective Magnetic Biochar Microspheres

**DOI:** 10.3390/ijms21113752

**Published:** 2020-05-26

**Authors:** Tao Zhao, Rongqi Chen, Junping Wang

**Affiliations:** 1 State Key Laboratory of Food Nutrition and Safety, Tianjin University of Science & Technology, Tianjin 300457, China; zhaotao1989@qlu.edu.cn; 2 School of Food Science and Engineering, Qilu University of Technology (Shandong Academy of Sciences), Jinan 250353, China; 3 College of Horticultural Science and Engineering, Shandong Agricultural University, Tai’an 271018, China; echo_chenrq@126.com

**Keywords:** biochar, Pickering emulsion, molecularly imprinting technology, magnetic

## Abstract

We report the use of biochar and Fe_3_O_4_ nanoparticles as co-stabilizers for oil-in-water (o/w) Pickering emulsion. The emulsion is subsequently used to prepare magnetic tetracycline-imprinted biochar composite microspheres (MMIPMs) with good uniformity and high selectivity. The MMIPMs were characterized by scanning electron microscopy (SEM), Brunner-Emmet-Teller (BET) measurements, Fourier transform infrared spectrometry (FT-IR), X-ray diffraction (XRD), vibrating sample magnetometer (VSM) and thermogravimetry analysis (TGA). The adsorption properties of tetracycline to the MMIPMs were investigated using different adsorption experiments including adsorption kinetic experiment, equilibrium binding experiment, selectivity evaluation and competitive adsorption tests. The theoretical maximum adsorption capacity of the MMIPMs (15.45 mg g^−1^) was greater than that of the raw biochar (2.10 mg g^−1^) and non-imprinted biochar composite microspheres (3.39 mg g^−1^) for tetracycline. Further, the MMIPMs were used as adsorbent for magnetic solid phase extraction (SPE) for the extraction of tetracycline present in drinking water, milk, fish and chicken samples. Under optimal conditions, the results showed good recovery yield ranging from 88.41% to 106.29% with a relative standard deviation (RSD) ranging from 0.35% to 6.83%, respectively.

## 1. Introduction

Tetracyclines (TCs), a large class of broad-spectrum antibiotic, have been extensively employed as therapeutic drugs and feed additives to fight infection and promote animal growth. Because of their unreasonable use and abuse, due to the recent increasing demands of livestock production, TCs have received increasing attention as an important class of contaminants of emerging concern (CEC). TCs pose potential hazards both environmentally and health wise, including the increase in multi-resistant bacterial strains, bioaccumulation and biomagnification in aquatic organisms, and can be carcinogenic to human beings upon long-term exposure [1,2,3,4]. Residual TCs could be absorbed by plants and livestock, and then transferred into human beings through the food chain, representing a major threat to human health. Therefore, it is of great significance to develop low-cost and efficient adsorption materials to remove tetracycline in the environment and food samples.

Biochar is a type of solid and carbon-rich material produced from the biomass pyrolysis under anaerobic conditions [5]. Biochar (BC) has received immense interest in environmental contamination remediation, such as agricultural and veterinary drug residues, environmental hormones, organic pollutants, soil heavy metals and so on [6,7,8]. Recently, Xian et al. reviewed biochar technologies in various wastewater treatments and summarized the future perspectives of biochar technology in wastewater treatment [9]. Wu et al. proposed an overview on soil remediation by biochar [10]. Rodriguez-Narvaez reviewed the biochar-supported nanomaterials for environmental applications [11]. Yaashikaa et al. reviewed the modification of biochar and the mechanism of pollutant adsorption using biochar [12]. Biochar, as an innovative adsorbent, shows potential positive aspects towards pollutant removal. However, the application of biochar-based adsorbent in the removal of environmental contaminants has left two problems unsolved: (1) The morphology of biochar depends on the biomass feedstock, and the messy morphology and size affect the separation efficiency of biochar-based adsorbent. The conventional preparation of spherical structured biochar include hydrothermal carbonization, a ball milling method and a sodium alginate/sodium silicate/chitosan assisted method [13,14,15]. However, spherical structured biochar prepared by hydrothermal carbonization has a low yield and poor particle size regulation. The ball milling method and the sodium alginate/sodium silicate/chitosan assisted method require expensive equipment and specific reagents. (2) Biochar is a broad-spectrum adsorbent that lacks specificity. Biochar modification is an important means to improve the specificity of biochar, including chemical modification and biochar nanocomposites. Chemical modified biochar could convert the weaker force between biochar and the target into a strong force (electrostatic effect, chelation, hydrophobic effect, etc.) through chemical modification. Biochar nanocomposites mainly involve the combination of biochar and functional nanomaterials [16,17,18], such as zero-valent iron (reducing), titanium dioxide (catalytic degradation), carbon nanotubes (aromatic structure) and ferric oxide (magnetic) etc. However, biochar is poorly dispersed in conventional solvents, which could cause biochar agglomeration during the chemical modification process, resulting in an uneven combination of functional groups/functional nanomaterials and biochar. Furthermore, the traditional preparation method of biochar nanocomposites usually requires harsh conditions. Therefore, it is of great significance to develop a versatile and mild method for preparation of biochar composite materials with tailored sizes and high specificity.

To solve the above two problems, we developed a Pickering emulsion polymerization method, combined with molecular imprinted technology (MIT) for preparation of magnetic biochar composite microspheres. The developed method has three advantages: (1) Pickering emulsion polymerization is a method for preparing spherical materials with regular morphology, which could change the emulsion polymerization conditions to regulate the morphology and particle size. (2) Biochar is well dispersed on the two-phase interface of Pickering emulsion, which could solve the problem of uneven chemical modification. (3) Pickering emulsion is compatible with a variety of functional nanomaterials, and the two-phase system of the emulsion can dissolve functional monomers with different polarities. In this work, we used the developed method for the construction of tetracycline-imprinted biochar/Fe_3_O_4_ composite microspheres (MMIPMs). The biochar particles were firstly treated with an alkaline solution to improve its hydrophilicity. The alkali-treated biochar and Fe_3_O_4_ nanoparticles were dissolved in distilled water as water phase, TC, methacrylic acid (MAA), divinylbenzene (DVB) and 2,2′-Azobis(2-methylpropionitrile) (AIBN) were dissolved in toluene as oil phase, and then the two phases were mixed and heated in a water bath. The obtained materials were separated by an external magnet, and the TC was eluted by Soxhlet extraction. The MMIPMs were characterized by scanning electron microscopy (SEM), Brunner-Emmet-Teller (BET) measurements, Fourier transform infrared spectrometry (FT-IR), X-ray diffraction (XRD), vibrating sample magnetometer (VSM) and thermogravimetry analysis (TGA). The adsorption properties were investigated via adsorption isotherm models, adsorption kinetics models, selective adsorption and competitive adsorption experiments. Furthermore, the MMIPMs were used for extraction of residual TCs in water and food samples.

## 2. Results and Discussion

### 2.1. Surface Properties of Raw and Alkali-Treated Biochar

**Figure 1:**
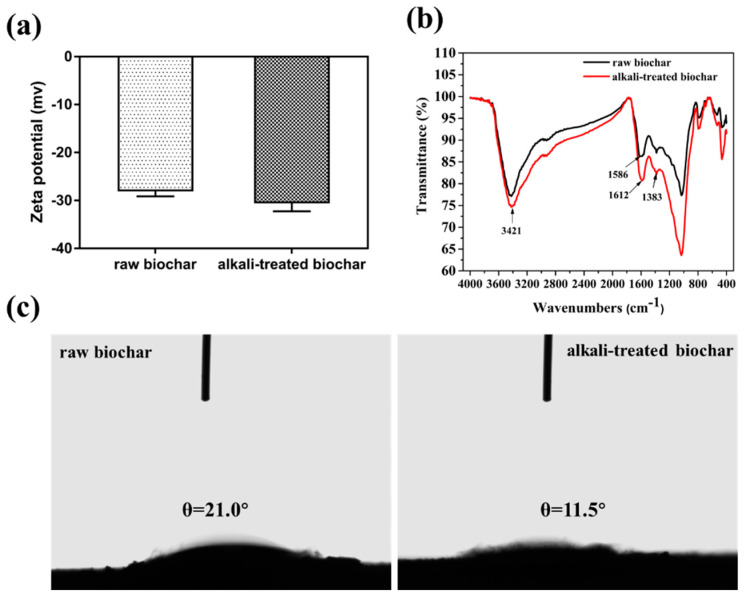
Zeta potential characterization of biochar before and after alkali treatment (**a**); FT-IR spectrum of biochar before and after alkali treatment (**b**); contact angle between biochar and water before (left) and after (right) alkali treatment (**c**).

The surface properties and functional groups of biochars before and after alkali treatment were evaluated. The zeta potential of the raw biochar increased from −27.3 to −32.3 mv after alkali treatment due to the exposure of basic groups on the surface of biochar (Figure 1a). This result is consistent with the results in the FT-IR spectrum, the peak intensity at 3421 cm^−1^, and 1612 cm^−1^ increased after alkali treatment, which were assigned to the -OH and the -C-O stretching vibration (Figure 1b). The contact angle between biochar and water decreased from 21.0° to 11.5° after alkali treatment (Figure 1c), the results indicated that alkali-treated biochar became more hydrophilic due to the increase of hydroxyl groups on its surface.

### 2.2. Characterization of MMIPMs

#### 2.2.1. SEM Characterization of MMIPMs

**Figure 2:**
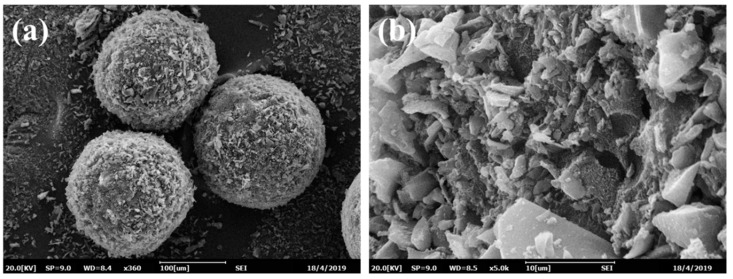
The SEM images of MMIPMs: Integral morphology (**a**) and surface morphology (**b**).

Figure 2a illustrates that the obtained magnetic tetracycline-imprinted biochar composite microspheres (MMIPMs) possessed a regular spherical structure and medium diameter distribution (70~100 μm). Figure 2b presents the MMIPMs with a rough surface composed of biochar particles. Pickering emulsion polymerization is a method for preparation of spherical material with armor structure that is composed of solid stabilizer particles. The results proved biochar as a new stabilizer for Pickering emulsion.

#### 2.2.2. Brunner-Emmet-Teller (BET) Measurements

**Figure 3:**
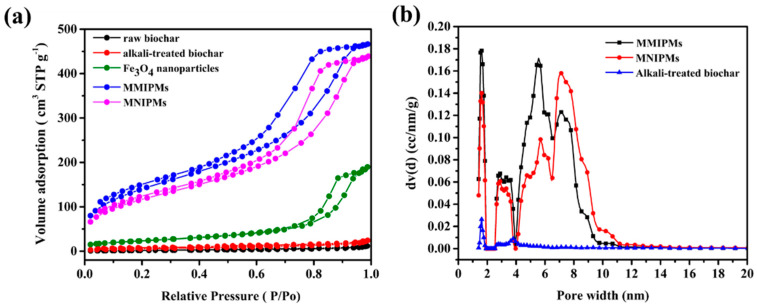
Brunner-Emmet-Teller (BET) measurements, N_2_ adsorption-desorption isotherms of raw biochar, alkali-treated biochar, Fe_3_O_4_ nanoparticles, MMIPMs and MNIPMs (**a**). Pore size distribution of MMIPMs, MNIPMs and alkali-treated biochar (**b**).

The N_2_ adsorption was carried out for raw materials and biochar composite materials (Figure 3a) in order to evaluate the permanent porosity. MMIPMs exhibit reversible type Ⅳ isotherm, which is one of the main characteristics of mesoporous materials. The hysteresis loop belongs to type H2b, which indicated the regular porous structure and uniform pore size of the MMIPMs. The pore size distribution of MMIPMs, magnetic non-imprinted biochar composite microspheres (MNIPMs) and alkali-treated biochar is shown in Figure 3b, the results indicated that the developed Pickering emulsion polymerization could significantly increase the number of mesopores of biochar. The surface area of MMIPMs was calculated to be 504.994 m^2^ g^−1^ using the Brunner-Emmet-Teller (BET) model, which is 67 times that of the raw biochar (Table 1).

#### 2.2.3. Magnetic Characterization of MMIPMs

**Figure 4:**
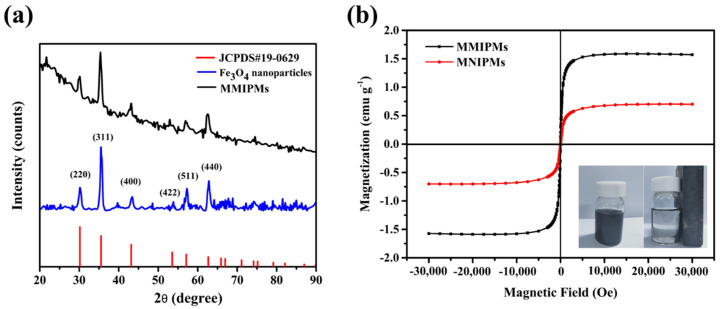
XRD spectrum of the synthetic Fe_3_O_4_ nanoparticles and MMIPMs (**a**), and magnetic saturation curve of MMIPMs and MNIPMs (**b**).

Magnetic characterization of MMIPMs was investigated by XRD and magnetic saturation curve, respectively. The results in Figure 4a indicated the diffraction peaks of the synthesized Fe_3_O_4_ at 2θ (30.24°, 35.59°, 43.25°, 54.20°, 57.29°, 62.88°) could be designated to reflections indexed to (220), (311), (400), (422), (511) and (440), respectively, which are consistent with the standard diffraction spectra of Fe_3_O_4_ (JCPDS card, No. 19-0629). Furthermore, the diffraction pattern of MMIPMs showed similar peak positions as that of Fe_3_O_4_, the results indicated that the crystalline structure of magnetite was well maintained during the synthesis process of MMIPMs. Moreover, the magnetic properties of MMIPMs particles were investigated, and the hysteresis loops and magnetic optical images were displayed in Figure 4b. The saturation magnetization of MMIPMs was 1.56 emu g^−1^, the MMIPMs were homogeneously dispersed in solution in the absence of a magnet, and could be completely separated from the solution in the presence of an external magnetic field (the lower right corner of Figure 4b).

#### 2.2.4. FT-IR Spectra and TGA Analysis of MMIPMs

**Figure 5:**
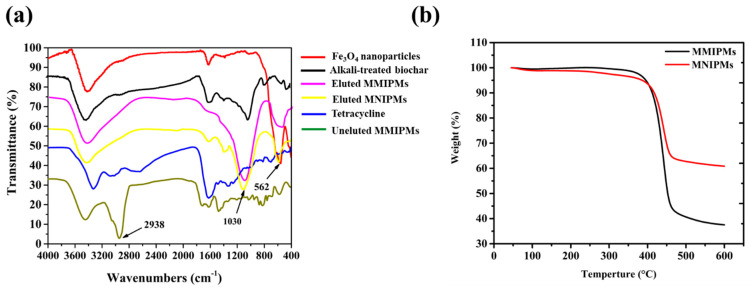
FT-IR spectrum of different materials (**a**), thermogravimetry (TGA) analysis of MMIPMs and MNIPMs (**b**).

The FT-IR spectra of Fe_3_O_4_, MMIPMs and MNIPMs are shown in Figure 5a. In the fingerprint area, the special peak at 1030 cm^−1^ may be assigned to C-O stretching vibration in the presence of phenols and hydroxyl groups of biochar, and the peak at 562 cm^−1^ could be assigned to Fe-O stretching vibration of Fe_3_O_4_ nanoparticles. The result indicated the MMIPMs were a composite of biochar and Fe_3_O_4_ nanoparticles. Furthermore, the peak at 2938 cm^−1^ of MMIPMs without elution could be assigned to TC, while no peak was observed at 2938 cm^−1^ of MMIPMs with elution, the results indicated that the template molecules (TC) were eluted clean during Soxhlet extraction. The thermal stability of MMIPMs and MNIPMs was estimated through TGA analysis from an ambient temperature to 600 °C at a heating rate of 10 °C min^−1^ under a nitrogen atmosphere, and the result is shown in Figure 5b. A significant weight loss occurred between 380–458 °C, which could be due to the breakdown of the material components. The results indicated that synthetic materials had high temperature resistance to decompose, which could meet the requirements of common experiments.

### 2.3. Adsorption Properties of MMIPMs on TC

#### 2.3.1. Adsorption Kinetic Experiment

**Figure 6:**
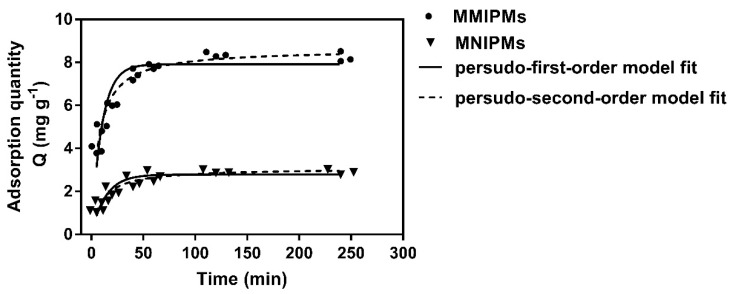
The kinetic adsorption data of MMIPMs and MNIPMs toward tetracycline (TC) were fitted with pseudo-first-order and pseudo-second-order models.

The results in Figure 6 indicate that the adsorption capacity of MMIPMs for TC increased with the adsorption time. The adsorption quantity reached equilibrium at 120 min, which was shorter than for traditional molecular imprinted polymers (equilibrium time, 8–12 h). The reason may be that the biochars on the surface of MMIPMs improve the porosity of the shell and improve the medium transfer rate of MMIPMs.

In order to investigate the mechanism of the adsorption of TC onto MMIPMs, the pseudo-first-order and pseudo-second-order models were employed to analyze the kinetic data. The curves and linearities of the two models and parameters were shown in Table 2. Compared to the pseudo-first-order model, the pseudo-second-order model was regarded as the better-suited model to describe the adsorption process referring to MMIPMs toward to TC due to the higher correlation coefficients (R^2^ = 0.975), as well as the coherence between experimentally obtained and theoretically calculated Q_e_ values. The pseudo-second-order model is suitable for reactions where saturated sites exist. The results indicated that the adsorption quantity of TC onto MMIPMs was dependent on the numbers of imprinted binding sites.

#### 2.3.2. Equilibrium Binding Experiment

**Figure 7:**
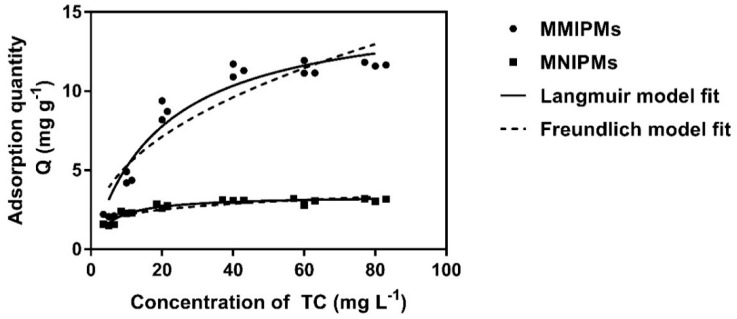
The equilibrium isotherm data of MMIPMs and MNIPMs toward TC were fitted with the Langmuir and Freundlich models.

Figure 7 represent the effect of the initial TC concentration on the adsorption performance and adsorption isotherms of MMIPMs and MNIPMs. It could be seen that the equilibrium adsorption capacity increased with the initial concentration of TC. The adsorption equilibrium of MMIPMs was achieved at the TC concentration of 60 mg L^−1^. However, for the equilibrium isotherm of MNIPMs there was no apparent adsorption equilibrium trend. The maximum adsorption capacity of MNIPMs (11.94 mg g^−1^) for TCs was 3.71 times as much as MNIPMs (3.22 mg g^−1^), the results indicated that the MMIPMs had good imprinting effects and identification performance.

Further, the Langmuir and Freundlich isotherm adsorption models were used to fit the experimental data. The results in Table 3 show that the Langmuir model fits well the sorption data with a correlation coefficient (R^2^) of 0.946 and 0.950 for MMIPMs and MNIPMs, respectively. The results indicated that the adsorption of TC onto MMIPMs was controlled by the chemical adsorption of imprinted sites on the biochar surface.

#### 2.3.3. The Selectivity Evaluation of MMIPMs

**Figure 8:**
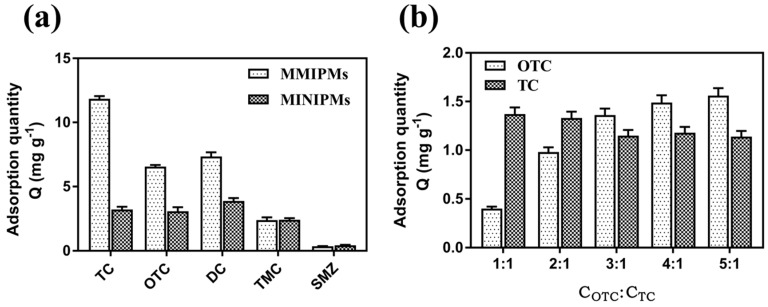
Adsorption capacity of MMIPMs and MINIPMs towards TCs (**a**). The competitive adsorption of MMIPMs toward OTC and TC (**b**).

The selectivity of the MMIPMs was investigated with oxytetracycline (OTC) and doxycycline (DC) as the structural analogues of the tetracycline (TC) template, and tsumacide (TMC) and sulfamethazine (SMZ) as reference compounds (Figure 8a). The results indicated that the MMIPMs had good selectivity toward TCs (TC, OTC and DC), compared with TMC and SMZ, and the MMIPMs with best selectivity towards TC among TCs (TC, OTC and DC).

To further investigate the selectivity of MMIPMs for TC, OTC was selected as a competitor for competitive experiment due to its similar molecular weight and structure. By increasing the concentration of OTC/TC to 3/1, the TC adsorption was decreased slightly. The results indicated that MMIPMs with high specificity for TC could effectively resist the interference of structural analogs during the adsorption process. The selectivity of MMIPMs for TC was attributed to the specific shape of the cavities produced in the imprinting process.

### 2.4. Real Sample Application

In order to verify the application of MMIPMS in real samples detection, four samples (milk, chicken, fish, water) were spiked with three TCs (TC and OTC) at three levels in real samples, and then extracted by magnetic solid phase extraction coupled to HPLC detection (Section 3.6). The recoveries for TC and OTC are shown in Table 4. The recoveries were 88.41~106.29% and 84.53~102.44%, respectively. The RSDs were 0.35~6.83% and 0.39~9.81%, respectively.

### 2.5. Discussion

In the past study, researchers focused on the improvement of adsorption performance of biochar, less attention was spent on the morphology of biochar. However, the mess morphology of biochar material affects its application in commercial packed columns and sophisticated analytical instruments. Pickering emulsion polymerization is a versatile method for preparation of spherical composite material with tailored sizes and good uniformity [19]. Furthermore, Pickering emulsion is a mild method for preparation of nano-composite materials, many functional nanoparticles have been reported as stabilizers for Pickering emulsion [20,21,22,23]. We used a gentle method to obtain versatile biochar composites microspheres by just adding functional monomers or functional nanoparticles into the biochar-based Pickering emulsion, such as hydrophilic/hydrophobic monomer, zero-valent iron (reducing), titanium dioxide (catalytic degradation) or carbon nanotubes (aromatic structure). These will be published in further studies.

In this manuscript, we used the developed method for preparation of highly selective magnetic biochar microspheres. More interestingly, the specific surface area of magnetic biochar composite microspheres (504.994 m^2^ g^−1^) was increased by 67 times compared with the raw biochar (7.467 m^2^ g^−1^). Therefore, Pickering emulsion polymerization is an effective way to increase the specific surface area of biochar. Molecular imprinting is defined as the construction of ligand selective recognition sites in synthetic polymers where a template is employed in order to facilitate recognition site formation during the covalent assembly of the bulk phase by a polymerization or polycondensation process, with subsequent removal of some or all of the template necessary for the recognition to occur in the spaces vacated by the templating species. As a result of Pickering emulsion being compatible with molecular imprinting technology (MIT), the problem of biochar specificity is effectively solved by Pickering emulsion polymerization combined with MIT [24,25]. The specific adsorption of biochar has been significantly improved through the developed method, which may be attributed to the improvement of specific surface area and the specific adsorption of imprinted sites.

## 3. Materials and Methods

### 3.1. Materials

Tetracycline (TC), oxytetracycline (OTC), doxycycline (DC), sulfamethazine (SMZ), tsumacide (TMC), methacrylic acid (MAA) and 2,2′-Azobis(2-methylpropionitrile) (AIBN) were purchased from Sigma-Aldrich (Shanghai, China). Iron trichloride (FeCl_3_) and ferrous chloride (FeCl_2_·4H_2_O) were obtained from Guoyao Group Chemical Reagent Co., Ltd (Shanghai, China). Divinylbenzene (DVB) was obtained from Rhawn Reagent Co., Ltd (Shanghai, China). Sodium hydrate (NaOH), Na_2_EDTA, methanol, ethanol, acetonitrile and formic acid were purchased from Sinopharm Chemical Reagent Co., Ltd (Shanghai, China). The straw biochar pyrolyzed at 400–450 °C was purchased from Liao Ning Golden Future Agriculture Technology Co., Ltd. (Anshan, China). All other solvents and reagents used in this study were of the highest available purity and at least of analytical grade. Doubly deionized water (DDW), obtained using WaterPro Water System (Labconco Corp., Kansas City, MO, USA), was used throughout the experiments.

### 3.2. Biochar Surface Modification

The raw biochar surface modification refers to relevant literature with minor modifications [6]. In detail: (1) The raw biochars were ground and then passed through a 70-mesh sieve to produce granules. (2) The granules (20 g) were then dissolved in 200 mL NaOH (3 mol L^−1^) solution and stirred for 2 h at 60 °C. (3) The obtained alkali-treated biochar particles were separated using a sand core funnel (G5) and washed with ethanol and distilled water until the eluent was at neutral pH value. (4) The alkali-treated biochars were subsequently dried in a vacuum oven (DZF-6020, BOXUN, Shanghai, China) at −0.1 MPa and 60 °C for 8 h and then stored in a dark and dry place.

### 3.3. Preparation of Magnetic Molecularly Imprinted Biochar Microspheres

Magnetic tetracycline-imprinted biochar composite microspheres (MMIPMs) were prepared by using Fe_3_O_4_ nanoparticles and alkali-treated biochar particles as co-stabilizers for Pickering emulsion polymerization.

#### 3.3.1. Preparation of Fe_3_O_4_ Nanoparticles

The magnetic Fe_3_O_4_ nanoparticles were prepared by a modified method. Firstly, 1.988 g FeCl_2_·4H_2_O and 3.244 g FeCl_3_ were dissolved in 100 mL distill water (the molar ratio of Fe^2+^ and Fe^3+^ is 1:2). The FeCl_2_/FeCl_3_ solution (5 mL) was added dropwise into 50 mL of 2 mol L^−1^ NaOH solution under vigorous mechanical stirring for 30 min at 80 °C. The Fe_3_O_4_ nanoparticles were separated under an external magnetic field, and then washed by distilled water until the elution solution turned neutral. The Fe_3_O_4_ nanoparticles were subsequently dried in a vacuum oven (DZF-6020, BOXUN, Shanghai, China) at −0.1 MPa and 60 °C for 8 h and then stored in a dark and dry place.

#### 3.3.2. Synthesis of MMIPMs/MNIPMs

90 mg alkali-treated biochar particles and 30 mg Fe_3_O_4_ particles were dispersed in 12 mL distill water and then sonicated for 10 min, the mixture solution was used as water phase. Then, 200 μL TC methanol solution (48 mg mL^−1^), 64 μL MAA, 278 μL DVB and 30 mg AIBN were dissolved in 1454 μL toluene as oil phase. The water phase and oil phase were mixed by hand-shaking for 3 min, and then reacted at 60 °C for 5 h until the stable spherical particles were formed. The resultant products were washed with methanol three times to remove the residual oligomers and monomers and filtered with a G5 sand core funnel. To remove the templates of TC, the obtained materials were washed with methanol/acetic acid (9:1, *v*/*v*) by Soxhlet extraction for 5 days, and then washed with methanol and distilled water (1:1, *v*/*v*) three times. The MMIPMs were dried in a vacuum oven at 60 °C. For comparison, the magnetic non-imprinted biochar composite microspheres (MMIPMs) were fabricated in same way without the molecular template.

### 3.4. Characterization of Materials

The surface properties of the raw, alkaline-treated biochars and MMNIPS/MNIPMs were performed with the contact angle measurement (OCA-20, Dataphysics Instruments Ltd., Germany) zeta potential (Zetasizer nano ZS 90, Malvern instruments Ltd., UK) and Fourier transform infrared spectroscopy (FT-IR) (Nicolet 6700 spectrometer, Thermo Nicolet, USA). The morphologies of MMIPMs were characterized by a scanning electron microscope (SEM) (EM-30 plus, COXEM, Korea) with an acceleration voltage of 20 kV. Prior to the observation, the specimens were vacuum filtered and golden-sputtered. The compositions of the Fe_3_O_4_ and MMIPMs were measured by X-ray diffraction (XRD) (D8-ADVANCE, BRUKER-AXS, Germany) with a 0.02° step from 10° to 90° in 2θ. The magnetic properties of the MMIPMs were measured by vibrating sample magnetometer (VSM) (MPMS-VSM, Quantum Design, America). The surface area, pore volume and pore size of MMIPMs and biochars were measured by nitrogen adsorption/desorption experiment (BET) (Autosorb-iQ, Quantachrome, Boynton beach, FL, USA). The stability of the MMIPMs was performed using thermogravimetric analysis (TGA) (TGA-1, Mettler, Switzerland). The heating rate was 10 °C min^−1^, with a nitrogen flow (99% N_2_) of 50 cm^3^ min^−1^, at temperatures ranging from room temperature up to 1000 °C.

### 3.5. Adsorption Properties of MMIPMs

#### 3.5.1. Adsorption Kinetic Experiment

MMIPMs (10 mg) were added to 5 mL TC methanol solution (20 mg L^−1^). Then the mixtures were mechanically shaken for different time periods (5 min, 10 min, 20 min, 40 min, 60 min, 120 min and 240 min) at room temperature. After that, the mixture was separated by an external magnetic field and the supernatant was immediately filtered through a nylon-66 membrane (0.22 µm) to remove suspended particles. The concentration of the TC in filtrate was determined by UV-Vis (λ_TC_ = 357 nm). The adsorption quantity Q (mg g^−1^) was calculated according to the following equation:

(1) $$Q=\left(C_{i}-C_{t}\right)V/m$$

where *C_i_* (mg L^−1^) represents the initial concentration of the TC methanol solution, and *C_t_* (mg L^−1^) is the equilibrium concentration of TC methanol solution. *V* (L) is the volume of methanol solution, while m is the mass of MMIPMs (g).

In addition, in order to evaluate the mass transfer and rate-controlling process, the pseudo-first-order (PFO) and pseudo-second-order (PSO) kinetic models expressed by Equations (2) and (3) were used to analyze the kinetics data of MMIPMs. 

The pseudo-fist-order kinetic equation:

(2) $$Q_{t}=Q_{e}\times \left(1-e^{-k_{1}t}\right)$$

The pseudo-second-order kinetic equation:

(3) $$Q_{t}=\frac{Q_{e}^{2}k_{2}t}{1+Q_{e}k_{2}t}$$

where *Q_e_* (mg g^−1^) and *Q_t_* (mg g^−1^) represent the equilibrium adsorption capacity, the binding quantity at different time *t* (min), respectively; *k*_1_ (min^−1^) and *k*_2_ (g mg^−1^ min^−1^) are pseudo-first-order and pseudo-second-order rate constants of rebinding processes, respectively.

#### 3.5.2. Equilibrium Binding Experiment

The static adsorption test was carried out to evaluate the adsorption capacity of the obtained MMIPMs/MNIPMs particles. In detail, 10 mg MMIPMs/MNIPMs were respectively added to 5 mL of different concentrations of TC methanol solution (5, 10, 20, 40, and 80 mg L^−1^). Then, the mixtures were mechanically shaken for 2 h at room temperature. Subsequently, the mixture was separated by an external magnetic field and the supernatant was purified by filtering with a nylon-66 filter membrane (0.22 μm). The TC solution before and after adsorption was detected by UV/Vis spectrophotometry to obtain the absorbance value. The binding quantity of MMIPMs/MNIPMs was calculated by Equation (1).

To further investigate the interaction between the MMIPMs/MNIPMs and TC, Langmuir and Freundlich isotherm adsorption models were applied to analyze the equilibrium binding data. The Langmuir and Freundlich isotherm adsorption models are expressed by Equations (4) and (5):

(4) $$Q_{e}=k_{L}Q_{m}C_{e}/(1+k_{L}C_{e})$$

(5) Qe=kFCe1n

where *Q_e_* (mg·g^−1^) and *C_e_* (mg·L^−1^) are adsorption quality and solution concentration at equilibrium, respectively; *Q_m_* (mg g^−1^) is the maximum theory adsorption capacity of the Langmuir model; *k_L_* (L mg^−1^) and *k_F_* (mg g^-1^(L·mg^−1^)^1/n^) are the adsorption constants of the Langmuir and Freundlich models, respectively; and *n* is the linearity index.

#### 3.5.3. Selectivity Evaluation

To evaluate the specificity adsorption of the synthesized MMIPMs/MNIPMs toward TC, another three compounds (OTC, DC and TMC) were studied for comparison. First, 10 mg MMIPMs and MNIPMs were separately mixed with 5 mL (40 mg L^−1^) solution of TC, OTC, DC, TMC, respectively. Then, the mixtures were mechanically shaken for 2 h at room temperature. After that, the mixtures were separated with an external magnetic field and the supernatant was filtered through a nylon-66 membrane (0.22 µm). The concentrations of six veterinary drugs were also detected by UV-Vis (λ _(OTC, DC, TMC)_ = 358, 356, 210 nm).

#### 3.5.4. Competitive Adsorption Tests

In order to further evaluate the specific adsorption of the MMIPMs toward TC, a competitive test was conducted by using OTC as the competitor, which has a similar molecular weight and structure to that of TC. The detailed approach is described as follows. A series of TC-OTC solutions with different concentration ratios (CTC: COTC = 1:1, 1:2, 1:3, 1:4, 1:5) were prepared by fixing the concentration of TC (5 mg L^−1^) and increasing the concentration of OTC. Then, 10 mg MMIPMs was added into 5 mL of the above TC-OTC solutions. After shaken for 2 h at room temperature, the polymer particles were removed from the suspensions by using an external magnetic field and the filtrate was obtained by filtration through a nylon-66 membrane (0.22 μm). The concentrations of compounds in the filtrate were detected by HPLC.

### 3.6. Extraction and Detection of Tetracycline in Water and Food Samples

#### 3.6.1. Sample Pretreatment

The fish and chicken samples were purchased from the local supermarket (Jinan, China), and the running water was taken from the laboratory. Five grams of fish sample was transferred to a 50 mL centrifuge tube. Then, 5 mL Na_2_EDTA-McIlvaine extract buffer was added and the mixture was then ultrasonicated for 20 min. The mixture was then centrifuged at 5000 rpm for 10 min and the supernatant was collected. The extraction process was repeated two times, and the extracts (10 mL) were collected for further use.

#### 3.6.2. The Optimization of Elution Condition

The impact of eluent types and volumes, and elution time on recovery were investigated. As can been seen from Figure S1, the optimal recoveries were obtained when using doubly deionized water containing 20% formic acid as eluent, 3 mL eluent and elution 20 min, respectively. Thereby, 3 mL of doubly deionized water containing 20% formic acid elute 20 was chosen for subsequent study.

#### 3.6.3. Application of Magnetic MMIPMs for Extraction of TCs from Samples

First, 20 mg MMIPMs was added into a volumetric flask and rinsed in sequence with methanol and water. Then, the MMIPMs were separated under external magnetic field and the supernatant was discarded. Subsequently, the sample extracts were added into the volumetric flask and the mixtures were mechanically shaken for 2 h at room temperature. After the extraction was complete, the MMIPMs were separated from the sample extracts under external magnetic field.

The MMIPMs enriched with TCs were washed with 2 mL water containing 1% methanol and then, the supernatant was discarded. The TCs were eluted from the MMIPMs by 3.0 mL aqueous solution containing 20% formic acid and detected by HPLC.

The main influencing factors including the type of eluent, volume of eluent and elution time were optimized, the performance of MMIPMs was assessed by the extraction recovery (ER):

(6) $$\mathrm{ER}\;=\;E_{i}/E_{0}\times 100\%$$

where E_0_ (mg·L^−1^) represents the initial concentration of the TCs in methanol solution, and E_i_ (mg L^−1^) is the concentration of TCs in the extracting solution after MMIPMs-SPE process.

#### 3.6.4. High Performance Liquid Chromatography (HPLC) Analysis

The detection of TCs was performed on an Agilent Technologies 1260 Infinity II HPLC (Agilent Technologies, Santa Clara, CA, USA). The HPLC column was a reversed-phase C_18_ column (250 mm × 4.6 mm, 5 μm, InertSustain). The mobile phase solvents were (A) methanol and acetonitrile (*v*/*v*, 3/7) and (B) 0.1% citric acid monohydrate aqueous solution (*v*/*v*, 21/79). The flow rate was 1.0 mL min^−1^. The column temperature was 35 °C. The total injection volume was 20 μL, and the TC was detected at 357 nm. The HPLC chromatogram of TCs standard solution and other real samples was shown in Figure S2.

#### 3.6.5. Method Validation

For comparison, the TCs in chicken and fish samples were detected by the standard method (GB/T21317–2007, China). The result showed that the recovery of TCs was 80.4~106%, and the RSD ranged from 1.9% to 5.7%. The results showed that the method established in this paper has good accuracy and precision.

## 4. Conclusions

In this study, we report a mild method for preparation of biochar nano-composite microspheres by Pickering emulsion polymerization combined with molecular imprinting technology. The magnetic tetracycline-imprinted biochar composite microspheres (MMIPMs) with good uniformity and high selectivity was prepared using biochar and Fe_3_O_4_ nanoparticles as co-stabilizers for a novel toluene in water emulsion. The specific surface area of MMIPMs (504.994 m^2^ g^−1^) was 67 times as much as the raw biochar (7.467 m^2^ g^−1^). The theoretical maximum adsorption capacity of the MMIPMs (15.45 mg g^−1^) was greater than that of the raw biochar (2.10 mg g^−1^) and non-imprinted biochar composite microspheres (3.39 mg g^−1^) for tetracycline, which was attributed to the chemical adsorption of imprinted sites on the biochar surface. Furthermore, the MMIPMs were used as a suitable absorbent for magnetic solid phase extraction to extract trace TCs, and its’ usability was tested and verified in water and food samples.

## Figures and Tables

**Table 1:** The surface area, pore volume, and pore width of raw materials and biochar composites materials.

Samples	Surface Area (m^2^ g^−1^)	Pore Volume (cm^3^ g^−1^)	Pore Width (nm)
Raw biochar	7.467	0.015	3.794
Alkali-treated biochar	21.070	0.032	1.614
Fe_3_O_4_ nanoparticles	84.091	0.276	9.333
MMIPMs	504.944	0.683	1.614
MNIPMs	421.435	0.644	7.124

**Table 2:** The fitting parameters of the pseudo-first-order and pseudo-second-order kinetic adsorption models for TC.

Samples	Kinetic Adsorption Models						
		Pseudo-First-Order Model			Pseudo-Second-Order Model		
	Q_e_(exp) (mg g^−1^)	Q_e_(cal) (mg g^−1^)	k_1_ (min^−1^)	R^2^	Q_e_(cal) (mg g^−1^)	k_2_ (g mL^−1^ min^−1^)	R^2^
MMIPMs	8.367	7.910	0.100	0.898	8.616	0.017	0.975
NMIPMs	2.913	2.784	0.073	0.911	3.084	0.032	0.952

**Table 3:** The fitting parameters of Langmuir and Freundlich isotherm adsorption models for TC.

Samples	Isothermal Adsorption Models					
	Langmuir Model			Freundlich Model		
	q_m_ (mg g^−1^)	k_1_ (L mg^−1^)	R^2^	K_F_ (mg g^−1^ (L mg^−1^)^1/n^)	n	R^2^
MMIPMs	15.450	0.051	0.946	1.942	2.307	0.861
MNIPMs	3.392	0.194	0.950	1.377	4.990	0.827

**Table 4:** Recoveries and relative standard deviations (RSDs) (%) obtained from the analysis of chicken, fish, milk and water samples spiked with TCs.

Samples	Analytes	10 μg kg^−1^		20 μg kg^−1^		40 μg kg^−1^	
		Recovery (%)	RSD (%)	Recovery (%)	RSD (%)	Recovery (%)	RSD (%)
Chicken	OTC	84.53	2.18	86.63	7.71	102.44	4.25
	TC	88.67	3.71	86.41	6.83	98.06	2.20
Fish	OTC	84.89	0.39	88.26	1.44	90.22	9.81
	TC	90.63	3.81	92.26	6.66	95.34	1.74
Milk	OTC	89.05	1.90	90.94	2.43	94.74	0.47
	TC	94.21	1.10	92.32	1.08	106.29	1.76
Water	OTC	92.01	0.47	91.38	2.81	93.92	3.17
	TC	91.91	2.34	96.45	2.12	95.68	0.35

## Supplementary Materials

The following are available online at https://www.mdpi.com/1422-0067/21/11/3752/s1, Figure S1: The optimization of SPE conditions: Eluent types (a), eluent volume (b) and eluent time (c). Figure S2: HPLC chromatogram of TCs standard solution (a), chicken samples (b), fish samples (c), milk samples (d), water samples spiked with 20 μg kg^−1^.
