# Supplementary material for: A Mild Method for Preparation of Highly Selective Magnetic Biochar Microspheres
Source: Int J Mol Sci. 2020 May 26;21(11):3752. doi: 10.3390/ijms21113752 (PMC7313027; doi:10.3390/ijms21113752)
Supplement: Supplementary file 1 [file ijms-21-03752-s001.pdf]

International Journal of *Molecular Sciences*

MDPI

Article

A mild method for preparation of highly selective magnetic biochar microspheres

Tao Zhao [1,2], Rongqi Chen [3], Junying Wang [4,*] and Junping Wang [1,*]

1. State Key Laboratory of Food Nutrition and Safety, Tianjin University of Science & Technology, Tianjin 300457, China
2. School of Food Science and Engineering, Qilu University of Technology (Shandong Academy of Sciences); Jinan 250353, China; zhaotao1989@qlu.edu.cn
3. College of Horticultural Science and Engineering, Shandong Agricultural University, Tai'an 271018, China; echo_chenrq@126.com
4. The Biotechnology Research Institute of Chinese Academy of Agricultural Sciences, No 12 Zhongguancun South Street, Beijing 100081, China

* Correspondence: wangjunying@caas.cn (Jy.W.); Tel.: +86-010-8210-9715 (Jy.W.); wangjp@tust.edu.cn (Jp.W.); Tel.: +86-022-6091-2484 (Jp.W.)

Received: date; Accepted: date; Published: date

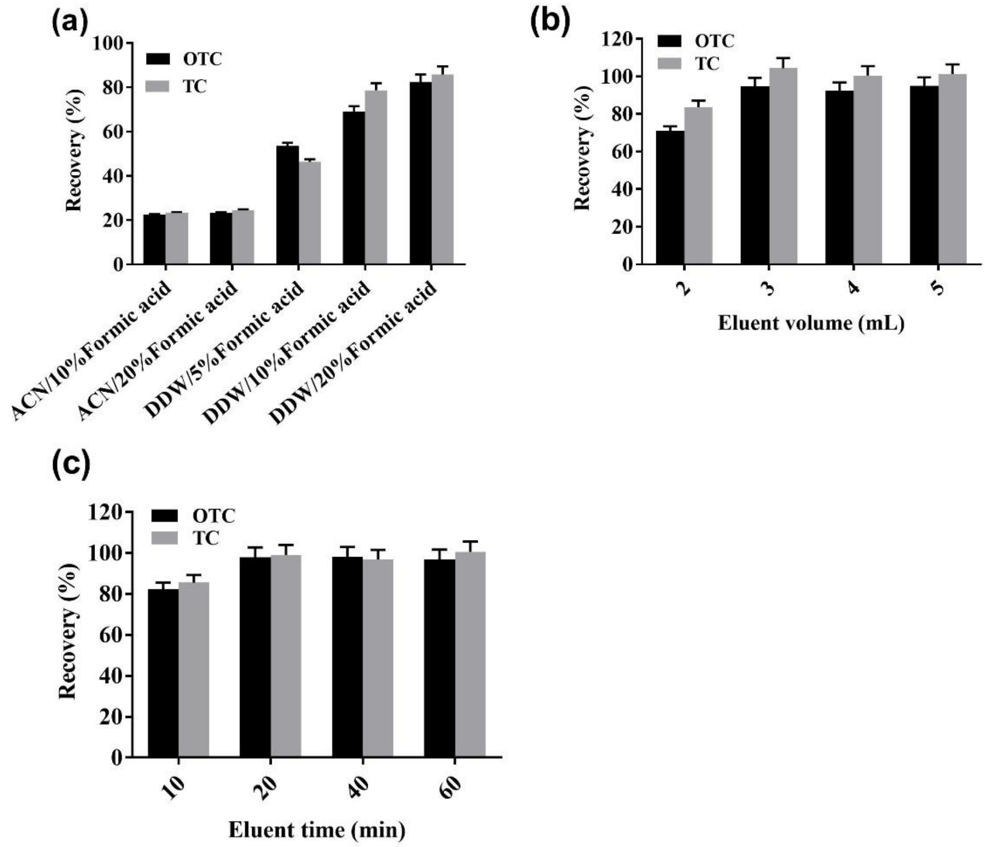

Figure S1. The optimization of SPE conditions: eluent types (a), eluent volume (b) and eluent time (c).

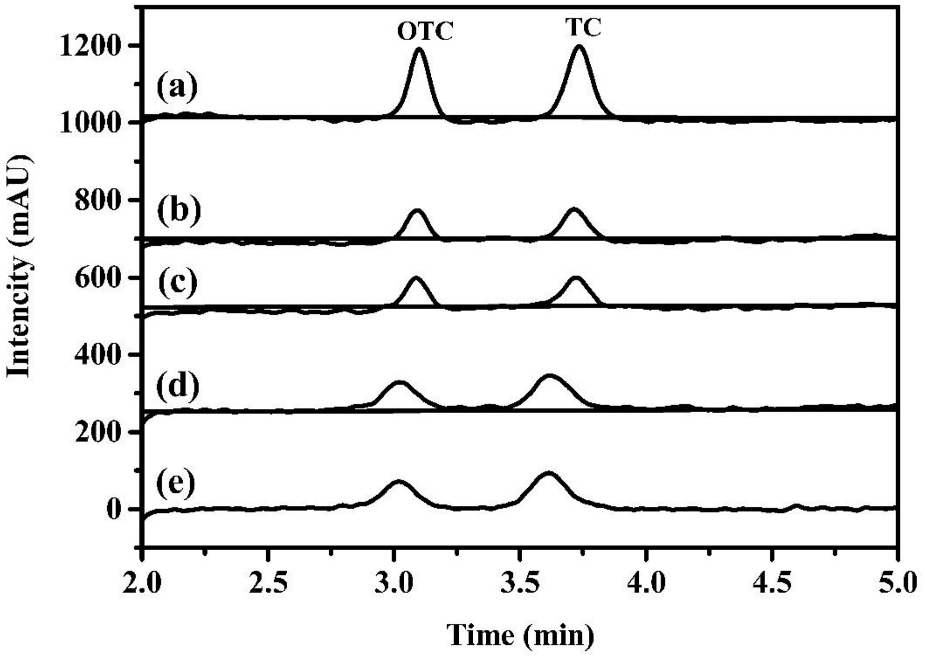

Figure S2 HPLC chromatogram of TCs standard solution (a), chicken samples(b), fish samples (c), milk sample (d), water samples (e) spiked with 20 μg kg^{-1}

© 2020 by the authors. Submitted for possible open access publication under the terms and conditions of the Creative Commons Attribution (CC BY) license (http://creativecommons.org/licenses/by/4.0/).
